# Employability of University Graduates with Disabilities in Spain

**DOI:** 10.3390/ijerph19031463

**Published:** 2022-01-27

**Authors:** María José Portillo-Navarro, Gabriela Lagos-Rodríguez, María-Leticia Meseguer-Santamaría

**Affiliations:** 1 Faculty of Economics and Business Administration, University of Murcia, 30071 Murcia, Spain; portillo@um.es; 2 Faculty of Economics and Business Administration, University of Castilla-La Mancha, 02071 Albacete, Spain; gabriela.lagos@uclm.es

**Keywords:** disability, employability, higher education, labor inclusion, social vulnerability

## Abstract

One of the most important challenges that Spanish universities face is the employability of graduates, a key factor to socioeconomic development. The analysis of the relationship between higher education and employment is a topic of obvious relevance, with greater interest in vulnerable groups—such as graduates with disabilities—who have a lower relative participation in university studies, a lower relative number of graduates, and lower employment rates. Thus, based on the Labor Insertion Survey of University Graduates (EILU-2019) by the Spanish National Institute of Statistics, this study addresses the influence of academic factors on the success of the employability of graduates in Spain, differentiating its effect in the case of people with disabilities. The results show the great importance of work experience during one’s studies and a knowledge of ICT to increase the employability and quality of employment among graduates with disabilities. This should become a key aspect of university training to reduce the economic vulnerability of this group.

## 1. Introduction

Labor market insertion is key to attain the resources to maintain a certain standard of living and, for that, education has always been a factor to be considered in the personal and social success of an individual. For decades, the education service has been one of the care benefits of the public sector, creating social mobility opportunities for those who start from a disadvantaged position. Although, ethically, education is a right for all citizens [1], one section of the economic doctrine ignores the individual and social “usefulness” of education. Since the mid-1960s until today, human capital formation and its impact on the economy has been a subject of particular attention.

Human capital theory [2,3] holds the belief that education increases individual productivity, which redounds to improved professional performance. Fundamentally, education of the individual allows them to acquire relevant competencies and skills for the work environment [4]. Within the framework of this theoretical construction, various theoretical and empirical studies have been carried out, including how these skills are adapted to the peculiarities and needs of each market [5], or the relevance of given factors, such as studying abroad [6,7,8,9].

Moreover, the job market signaling theory [10,11] stresses the inability of employers to value, in a rational way, the competencies of their potential employees. The existence of uncertainty and asymmetry in the information available to the parties makes the employment relationship an area in which to apply the principal–agent relationship. Thus, the employer, who does not have certainty about the real abilities or competencies of the potential employee, takes certain signs or signals as their criteria of choice and, among them, this includes the educational level of the individual. In this way, academic accreditations become a mere indication of the skills of the future employee, and therefore, productivity is a matter inherent to the individual, rather than relative to his or her level of education.

With these two theoretical conceptions serving as framework, the numerous empirical studies on the relationship between education and the labor market have focused on the analysis of the educational factors that affect the employability of individuals [9,12]. The results are not conclusive, and employers often make different assessments of individuals with a similar educational level, based on their own value system [13].

All these issues also affect persons with disabilities, who, in addition to the general difficulties in finding employment, have other issues related to their disabilities and the stereotyping of them in the labor market. Thus, the lower employability of persons with disabilities translates into a source of economic vulnerability and self-esteem. Hence, it is necessary to continue to investigate labor market insertion of graduates, as well as the variables that influence it. This issue, although previously addressed by other authors such as [14,15,16], has not been analyzed in sufficient depth for persons with disabilities.

Therefore, this paper addresses the analysis of the labor market insertion of graduates, with and without disabilities, considering a series of variables which have an impact on labor market insertion, such as employment situation and stability, wage level, adequacy of training to employment, or working hours.

To this purpose, after presenting the theoretical framework, a statistical analysis of the data provided by the latest Labor Insertion Survey of University Graduates (EILU) [17] is addressed. Using binary logistic regression models, the effects of educational factors on the variables of success in labor insertion are estimated, for graduates without and with disabilities, showing the different impact on both groups and which aspects should be influenced to reduce the risk of economic vulnerability. The conclusive results are included in the conclusions section of this paper.

## 2. Theoretical Framework: Higher Education and the Labor Market

It is undeniable that education provides students with skills and increases their innate qualities. However, it is essential to consider the validity that employers award to education.

The most recent studies have focused on what criteria employers use to decide on the recruitment of their employees, and this concern is specifically aimed at trying to adapt tertiary education to the demands of the labor market, [14,18,19,20,21,22,23]. This is not an easy matter, as not only do employment needs change by country, but also the choice of the employer will be conditioned by the socio-economic, political, and cultural peculiarities of the environment. The scientific contributions of this nature are inscribed in the institutional theory, and approach the labor insertion in a context of interdependence among individuals, institutions, and the norms that regulate them [24,25].

These aspects, inherent to employment search and labor market insertion, are decisive for persons with disabilities too, since they are in a particular situation that requires a specific analysis, such as the one carried out in the present paper.

### 2.1. Employment and Labor Market Inclusion of University Students

Education is considered crucial for the working life of individuals and the social and economic development of a country in any of the abovementioned theoretical approaches. Governments and international organizations have therefore promoted studies and research aimed at improving training, and thus ensuring the proper functioning of the labor market [26,27].

Nowadays, a university does not only transmit or generate knowledge: it also guides the training of its students towards employment [28]. In fact, the level of success in entering the labor market serves as an indicator of the quality of education received, particularly at university level [29]. This author identifies universities and students that have been inclined to respond to the needs of the labor market when making teaching and learning decisions.

Nevertheless, the environment they face is uncertain due to increasing economic globalization and the emergence of information and communication technologies. The introduction of technology is generating important and rapid changes in the employment systems, owing to the obsolescence of certain traditional jobs and the emergence of new ones. Therefore, technology is a variable to consider regarding labor market insertion. To adapt to this situation, employers’ expectations have come to be a benchmark in the design of university education provision, and not only in terms of degrees and curricula. There is also a special interest in certain factors, such as knowledge of other languages, which may be important for the success of graduates, and have an impact on entering the labor market. Accordingly, the adaptation of training to labor market needs is one of the elements to be considered.

There are two issues that must be differentiated in considering the insertion of Spanish university graduates into the workforce. On the one hand, the employability derived from their studies, that is, the acquisition of specific and transversal competences which qualify them for the professional activity related to their studies [22], and, on the other hand, the effective achievement of quality jobs connected to their specialization. Both aspects are key to determine the efficiency of public activity, especially in a country such as Spain, where almost 80% of students are enrolled in a public university.

It is necessary to compare the results obtained to the financial effort made, both because of the volume of public resources devoted to the provision of university-level education (according to OECD [30] data, Spain spends 4.3% of its GDP on education), and because of the impact of the formation of human capital on the Spanish economy. The employability of graduates is one of the indicators used, since it is a constant concern for universities [22]. Consequently, analyses on labor market insertion become indispensable [15] in addition to those factors which impact success in labor market insertion.

Data on higher education graduates in Spain places this country on the OECD average, and slightly above the European Union average [31], with its annual upward trend being relevant. In fact, the Spanish population aged between 24 and 29 who have completed higher education is 50.2%, the majority (34.6%) of which are university graduates [32].

The impact of higher education on the Spanish labor market should be positive, and in line with the goal of the U.N. [33], which specifies that productive employment and decent work are key factors in achieving fair globalization and reducing poverty. Baquero and Ruesga [17] point out that lower income social strata should relate to access to university education, because tertiary education is conceived as a tool to reduce inequality. Moreover, there is no doubt that disability is a factor of inequality, and that university education should contribute to amending this. According to INE (Spanish National Statistics Institute) [32] data for the second quarter of 2021, the unemployment rate for people with primary education was 26.35, while for graduates it was 9.52. In the 25–29 age group, unemployment rate among university graduates was 16.39, far below that of those with the first stage of secondary education (30.51). The latest available data [34] show that, in 2018, approximately 1.5% of university students in Spain were people with disabilities, mainly with physical disabilities (55.9% of the total).

In this paper, we aim to analyze if the Spanish higher education system, in addition to contributing to the employability and the labor market insertion of university graduates, ensures its purpose of correcting inequities between people with and without disabilities. In other words, we want to identify whether the university training offered in Spain contributes significantly to the improvement of opportunities for those more disadvantaged groups. In particular, this paper aims to identify the influence of the educational system on the success of the labor market insertion of Spanish university students with disabilities, and the differences within the whole population.

### 2.2. Factors for Success in Employability of University Graduates

Studies on employability and labor market insertion highlight certain dominant factors in their explanations. Some of these factors have to do with the socio-political and economic context of the moment (for example, ref [35] analyzes the impact of the 2008 financial crisis on the labor market insertion of graduates, focusing on the problem of over-qualification) and others with the own characteristics of the graduate, derived or not from their own decisions. The European Commission states [26] there are particular types of factors, such as age, gender, ethnic group, disability, or social class, which can alter the opportunities to enter the labor market. These kinds of circumstances can lead to discriminatory practices that are generally omitted when considering the employability of these types of graduates. Specifically, in the subject of disability, ref [36] points out that there is a disadvantage in entering the labor market.

The efforts of universities to improve the employability of their graduates have led to the incorporation of several of these factors into the university activity. This is the case with the facilitation of internships offering professional experience to the student, the teaching of other languages, or the facilitation of international exchange programs. In this paper, we will take as reference some of those external factors that can determine the so-called success in the labor market insertion, including the condition of disability. The aim is to identify whether the impact of each factor is the same for graduates with and without disabilities, and to assess said impact as an explanation of their success in entering the labor market.

One of the factors to which more attention has been devoted is the work experience of the graduate. As [14] points out, this is a discussed issue where there is evidence of both negative effects—e.g., there is less time to study [18], although Ruesga et al. [37] did not find a significant negative impact on academic achievement if the workload was not excessive—and positive effects, such as resulting in a higher salary level for university graduates [38]. Neyt et al. [39] made an in-depth review of the literature on the subject and concluded that the negative effects were more intense during the decision to start or continue their studies—especially for university students—rather than in the student’s performance during their studies.

Another factor that has been analyzed is whether the graduate has been granted a scholarship [28]. In this case, the results vary according to the type of the scholarship, with the impact of scholarships being greatly associated with the excellence of the student, and less associated with scholarships that are linked to other purposes, such as the economic provision of services in the university itself.

In a global environment, the importance of languages as a factor of employability and labor market insertion is evident. Aware of their relevance, universities have integrated knowledge of languages other than the mother tongue as an additional element to obtain a university degree. Many studies, especially in postgraduate studies, are already taught in other languages. National and international studies support employers’ appreciation of graduates’ ability to speak and write in another language [40].

The technological knowledge of the future employee is another of the factors most valued by employers [41], resulting in greater labor market insertion for those with a more advanced level [28].

All these factors can influence the success of labor market insertion. Consequently, it becomes a priority to determine which factors are more significant or have a greater impact on labor market insertion.

### 2.3. Employability of University Graduates with Disabilities

People with disabilities face specific problems in attempting to enter the labor market. These barriers include accessibility, discrimination, education, participation, and social protection [42,43,44]. In particular, discrimination can affect decisions regarding their education and the time and investment they want to make in it [45].

Thus, in recent years, both at a European Union and at a national level, the National Action Plans for Employment have been developed as result of the change in the direction of disability policies, from being more focused on subsidy, income transfers and sheltered employment, to the realization of an employment policy as an essential element for the achievement of social integration [46]. This has led to the development of active employment policies to reduce the gap between employees with and without disabilities. This is the result of social integration being one of the objectives of modern societies, where including everyone, with an appropriate management of diversity inherent to the concept of citizenship, has led to an important change in the perspective on people with disabilities. They have come to be considered an active part of the society rather than only citizens that receive aid and subsidies [47,48,49]

Furthermore, it is necessary to acknowledge that people with disabilities who work in a company or institution may represent a valuable contribution, provided that the entity in which they carry out their work is able to make appropriate use of their competencies through suitable human resource management to develop a positive and proactive strategy for the management and integration of disability in the workplace [50]. Progress has been made to align diversity management actions with the strategic approach of organizations, both at an international level (such as [51]) and at a national level (for example, ref [52], prepared by the Spanish Ministry of Labor and Social Economy) [53,54,55,56].

However, it is undeniable that, at present, there are significant differences in labor market insertion for people with and without disabilities and that there is still a long way to go. The need to address the analysis of factors influencing the labor market insertion of people with disabilities becomes a priority to be able to respond to the current situation and to improve understanding of reality, providing different points that can serve to improve the labor market insertion of this group.

For this analysis, some relevant data to contextualize the current situation—extracted from [52] prepared by the Spanish Ministry of Labor and Social Economy—would be that people with disabilities, in 2019, represented 6.2% of the working age population, a percentage that has been increasing in recent years, compared to 2015 when it was 5.9%.

Focusing on the group of people of working age with disabilities, the most recent data show a great difference regarding gender: 56.8% are men and 43.2% are women. This difference is even greater in the section of people under 25, where men account for 64.2%, and women for 35.8%. By education level, people with disabilities with secondary education account for 56.7%, while people without disabilities account for 58.8%, making them close values. However, in the area of higher education, the gap is much greater, whilst people without disabilities, of working age with higher education, are 36.7%, while those with a disability represent 16.7% [52]. According to data from the National Institute of Statistics [57] in 2019, 29.1% of people with disabilities were employed accordingly to their level of education (they did not have an educational level higher than that required in the workplace). These values lead to a need to analyze which variables impact labor market insertion of this group of people, since although employment is important for everyone, in the case of people with a disability, independence and social inclusion are favored. This also opens opportunities and social connections, improves self-esteem and the possibilities of maintaining a situation in which family dependency can be reduced [58].

Some of these variables, such as income level or salary, have been examined in various studies [45,46], finding evidence that workers with a disability earn less than workers without a disability. Nevertheless, these variations may be determined by differences in productivity between the two groups, which would have an impact on the salary, thus explaining the variations. Therefore, some studies—such as [59]—have addressed this issue, although the results have not been conclusive in this regard.

The recognition of the need to support people with disabilities and to make a greater effort for their labor market insertion has recently led to the elaboration of a future Spanish Disability Strategy 2022–2030 to build a roadmap that can help this group and their families, as has been performed at a European Union level.

After this brief characterization of the labor market situation of people with disabilities, success in entering the labor market may be determined by the same variables as for people without disabilities, such as employment situation and stability, wage level, and adequacy of training to employment or working hours. First, it is necessary to analyze, according to the available data, whether the disability is a differentiating factor or not in terms of success in the labor market insertion, and whether the variables that affect graduates to achieve such success equally influence students with a disability.

## 3. Materials and Methods

To achieve the main objective of this research, the need to identify whether disability is a differentiating factor in the employability of graduates in Spain was first addressed. Following the model established in [14,15], the factors that influence the success on entering the labor market and how they influence it were analyzed, drawing a distinction between graduates with and without disabilities.

The Labor Market Insertion of University Graduates Survey (EILU 2019) by the National Institute of Statistics [17] was used. The aim of this survey was to analyze the different aspects of the labor market insertion of university students.

The monitoring of university graduates provides relevant information for students’ decisions when they choose the degree or master’s degree they want to pursue, as well as for teachers and Public Administrations, who design educational policies. We looked for a study that could assess the influence of university education on the process of job insertion, and provide data on this transition, and its quality, that would allow us to measure its success.

The chosen survey was a continuous four-yearly survey. The 2019 edition was the second wave, with the first dating back to 2014.

The population scope was made up of university graduates (including first and second cycle graduates) and master’s degree graduates from the Spanish university system. The survey was targeted to graduates from Spanish universities in the 2013–2014 academic year, considering that it takes about three years from completion of studies to stabilize the relationship with the employment world. Thus, the collection of data was developed in the period between July and December 2019.

Its territorial scope extended to all universities in Spain. The reference period for the results was from July to December 2019, and the corresponding period for the information was the period between the end of university studies and the time of the interview.

There were two types of information: objective information (qualification obtained, first job, current occupation, time elapsed to find a job, etc.), for which administrative sources were used, and subjective data (suitability of studies to employment, difficulties in finding employment, skills acquired, reasons for geographical mobility, etc.) for which the survey was used directly. Therefore, a combined method of data collection was used: registers and direct surveys.

The administrative sources of information were: the Integrated University Information System (General Secretariat for Universities), the files on affiliations and contribution bases (Social Security), the files on contracts and occupational training (Public Employment Service), the State Database for Persons with Disabilities (IMSERSO-CERMI-MSCBS), and the National Register of Persons with Disabilities of Inhabitants and the Register of Spaniards Living Abroad (National Institute of Statistics), all official sources.

The sample size was 31,000 university graduates, and 11,000 master’s degrees.

Regarding sample design, two populations were targeted: university graduates and master’s graduates. The sample frame was for graduates and graduates of master’s degrees at Spanish universities in 2013 and 2014. Each record contained the identifying variables, the variables that defined the levels of disaggregation for the estimates, and additional information on date of birth, sex, and nationality. This type of sampling was mono-stage without replacement and with equal probabilities, and the selection was made in each of the estimation domains considered. Thus, a simple random sample was taken from each autonomous community of the University in which the students were graduated, as well as the records of each cell by variables such as field of study, gender, type of university and field of study, which defined the other levels of disaggregation. Finally, the sample was adjusted so that all classifications had a sufficient sample.

Data collection was carried out in two stages, via the Internet (CAWI) and by telephone (CATI). The work was designed in eight blocks to facilitate study.

In the first place, the success in labor market insertion was identified through the set of independent variables indicated in the model [14,15], referring to the time of the survey (2019). The separated analysis of these variables provided the measure of the quality of the employment found by the students in their incorporation into the labor market and allowed us to see the weight that each variable had on the result.

Next, the possible external factors that may have conditioned the success of labor market insertion were identified, and the impact of disability was established as an important element in this process. Subsequently, the studied population was divided regarding their disability condition, and a comparison was drawn on how they were affected by the rest of the external factors.

Given the dichotomous nature of the dependent variables studied, a binary logistic regression model was chosen, its elements were:

(a) Dependent variables, to quantify “successful labor market insertion”:
  - Employment situation (EMP_SIT): currently working (value 1) or not (value 0);
  - Employment stability (EMP_STA): having an indefinite contract, or being an employer (value 1), versus having a temporary contract (value 0);
  - Income quintile (INC_Q): have a salary that falls within the highest 40% (4th and 5th quintiles) on the basis of Social Security contribution (value 1), versus being within the lowest 60% (value 0);
  - Adequacy (EMP_ADE): working in a job that requires a university education (degree, master’s degree, or doctorate). Takes the value 1 if they are, and 0 otherwise;
  - Working hours (WOR_HOU): working full time (value 1) versus part time (value 0).
(b) Educational factors:
  - Work during studies (WORK): value 1 if he or she worked during university, and 0 otherwise;
  - Has been granted a scholarship (based on their academic merits) during studies (SCHOLARSHIP): value 1 if granted, and 0 otherwise;
  - Has undertaken part of their studies abroad (MOBILITY): value 1 if he or she did, and 0 otherwise;
  - Level of knowledge of information and communication technologies (ICT): divided into three categories: basic, intermediate, and advanced. The analysis was performed by taking the first variable as a reference;
  - Number of languages in addition to mother tongue (LANGUAGES): categories are: 1, 2, 3, 4 and 5 or more.
(c) Socio-demographic factors:
  - Gender (GENDER): takes the value 1 for women, and 0 for men;
  - Age group (AGE): divided into three groups: up to 30 years, 31 to 34 years, and 35 years or older. The analysis was performed by taking the first variable as a reference;
  - Disability (DISAB): two groups: graduates who have been granted at least 33% disability and those who have not. Takes the value 1 if they have, and 0 otherwise.

## 4. Results

**Figure 1:**
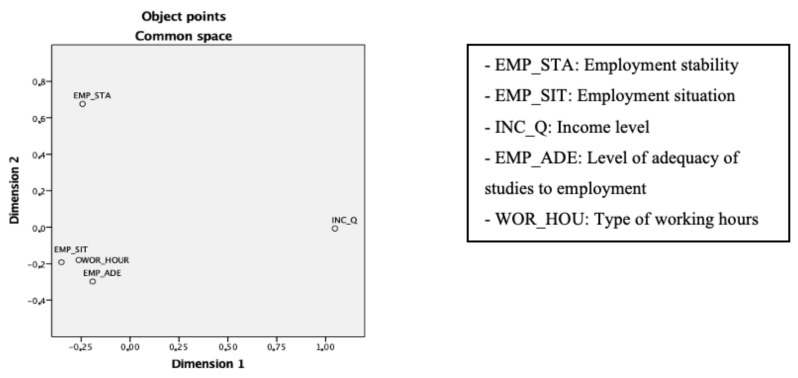
Own elaboration based on data from the Spanish National Institute of Statistics.

Success in entering the labor market was measured by the variables already mentioned (employment situation, employment stability, income level, level of adequacy of studies to employment and type of working hours). By means of a multidimensional scaling, it was justified that these items presented differentiated behaviors and the relationship between them, represented graphically in the following Figure 1.

As can be seen, by reproducing the distances between the variables (S-stress = 0.00066; Tucker’s congruence = 0.99986), in the first dimension, the income quintile had a clearly differentiated behavior, which should result in a different impact on the explanatory variables, showing that the factors that determine the level of salary of graduates would not affect them in the same way as the rest of the variables of employment success. The second dimension separated employment stability from the other three variables, so that factors were also expected to have a different structure (of significance and/or intensity). With respect to the other three variables, although they had similar correlations, they measured different aspects; therefore, it was interesting to keep them separate.

These five variables evolved differently between 2014 and 2019 (the two waves of the EILU survey), with different behaviors between graduates with and without disabilities, as shown in Table 1 below.

Variables related to job situation and stability showed relative improvements between 2014 and 2019 for graduates without disabilities (percentage of employed people increased from 80.8% to 86.2%, and the percentage of people in permanent employment rose from 46.3% to 58.2%), which did not occur among graduates with disabilities (dropped from 75.7% to 75.4% for employed people, and there was a smaller increase from 46.4% to 53.2% for permanent employment).

The percentage of graduates with a relatively high level of income fell significantly (from 42.3% to 30.3%), with a more pronounced decline among graduates with disabilities (from 41.9% to 26.8%). Analyzing the degree of adequacy between the job and having a university degree, the percentages of graduates in a job with this requirement fell in both groups (from 74.8% to 68.3% among those who did not declare disabilities, and from 68.2% to 63.3% among graduates with disabilities). Finally, the percentage of graduates with a full-time job among persons with disabilities fell sharply (from 78.7% to 64.5%), while the other group remained almost stable (from 76.5% to 74.3%).

In summary, the indicators of success of labor insertion were not only lower for graduates with disabilities, but their evolution has also been worse since 2014, increasing the economic vulnerability of this group.

With the aim of identifying the incidence of disability in the success of labor market insertion of university graduates in Spain, logistic regression of the dependent variables identified on each of the factors was carried out. We also analyzed how disability behaves as an influencing factor, with the other factors acting as control variables. The results are shown in Table 2.

It was noted that all control variables were significant for the employment situation, except for having received a scholarship that was based on academic merits.

Analyzing disability, the data indicated that having a disability had a negative impact on all the characteristics which we defined as relating to success in labor market insertion. Graduates with a disability were half as likely to be working as those with no disability (Exp(B) = 0.504), their employment stability was reduced by about 25% (Exp(B) = 0.736), as was their salary (Exp(B) = 0.762), also the adequacy of their training to the job was worse (Exp(B) = 0.882), and finally, they were 40% more likely to work part time (Exp(B) = 0.607).

Once it was established that disability was a condition influencing the entry of university students into the labor market, we studied how other factors intervene in labor market insertion, differentiating between graduates with and without disabilities in each of the dependent variables. The results are shown in Table 3.

Generally, external factors are significant with respect to the characteristics that identify success in labor market insertion, both for students with and without disabilities. However, this is not always the case. Subsequently, the significance and influence in the employment integration was analyzed, factor by factor.

Regarding the first factor, the fact that graduates worked during their university studies was significant in both groups, and increased the chances of professional success, although in people with disabilities this impact was much greater. For example, the odds ratio of working at the time of the interview in the case of graduates with disabilities multiplied by 6 (Exp(B) = 6.074), and only doubled (Exp(B) = 1.974) in the case of those not having a disability.

Having received a scholarship was not significant in several cases: in the working hours for the group with disabilities, and in the employment situation and salary for the group of people without disabilities. Its effect was practically nil among graduates without disabilities.

Graduates who undertook part of their studies abroad improved their chances of a successful labor market insertion, especially among the group with disabilities.

The gender factor functioned differently for people with and without disabilities. While, for the former, being a woman was an advantage to find a better position in the labor market, for people without disabilities, the situation was the opposite. It was noted that for graduates with disabilities this was not significant in employment stability.

The age analysis showed that, for people with disabilities, the younger the graduates, the more likely they were to improve their employability. However, for graduates without disabilities, the effects of age were not significant.

Regarding the last two factors, which referred to the level of skills acquired in relation to technologies and languages, it was noted that this favored a successful entry into the labor market for graduates, more clearly in those with disabilities. Regarding languages, data indicated that the influence was low, since the Exp(B) were very close to 1.

To study the importance of educational factors to differentiate (or predict) the values of each of the variables of success in job insertion among graduates with disabilities, the structure matrices were obtained (pooled within group correlations between discriminating variables and standardized canonical discriminant functions).

As Table 4 shows, the factor with the greatest ability to predict success in employment insertion was having worked during one’s studies. This shows the importance of a university education combined with work experience, both for job insertion (EMP_SIT) and for job quality, since it was the factor that best predicted having a permanent job (EMP_STA), full-time job (WOR_HOU) and a high salary (INC_Q); it was also the second factor for having a job related to the studies completed.

The second relevant educational factor in the employment situation of graduates with disabilities was related to ICT skills. This also had high correlations for full-time and education-related employment (where this is the most relevant factor); however, the ability to predict high wages and full-time employment was reduced.

Having completed part of one’s studies outside Spain (mobility factor) had a low predictive capacity for the job situation of graduates and for the adequacy of their employment to the studies completed, being irrelevant for stability, salary level and working hours.

Finally, the scholarship and languages factors did not have the ability to predict job insertion success in any of their five variables.

## 5. Conclusions and Limitations

The success of graduates in entering the labor market is a decisive factor in their emotional, economic and social vulnerability, and education plays an important role in this, being an essential pillar for personal and professional growth. This success can be measured through the analysis of the behavior of the variables: employment situation, employment stability, income level, adequacy of training to employment and working hours.

The success of the labor market insertion of graduates has been addressed on numerous occasions, although few studies have analyzed this variable regarding disability. In this paper, we delved into the analysis of the factors that influence the success of the labor market insertion of graduates with disabilities, compared to those without disabilities. Research supports the notion that the condition of disability influences this success in Spain, as well as the influence of each of these factors in each case.

The overall picture shows that students with disabilities have a much lower presence in university studies compared to the non-disabled population, an inequality that remains in access to the world of work. Since both training and access to quality work give people key independence for their personal and social development, research is needed into how these differences occur, and what actions need to be taken to reverse this situation.

Upon entry into the labor market, graduates with disabilities face additional problems due to their disability status, as they may require certain job adaptations in order to perform effectively. In order to alleviate this disadvantage and promote social diversity, more active labor policies have been adopted, aimed at social and labor integration, abandoning policies based on subsidies, sheltered employment or income transfers.

Ultimately, the mindset is shifting towards a more diverse society in which we are all actively contributing, and understanding that, in so doing, the society becomes richer.

For university graduates, working while pursuing university studies, having received scholarships based on academic merits, having undertaken part of their studies outside of Spain, gender, age, level of knowledge of ICT, and the number of languages known, are factors which influence a successful insertion into the employment world.

These factors have different effects when we distinguish between students with and without disabilities, causing their labor market insertion to be limited by the condition of disability they may have.

A successful labor market insertion is favored when graduates have worked during their studies, have completed part of their studies outside of Spain, or have a high level of ICT knowledge, especially among those with a disability. However, the fact of having received a scholarship based on their academic merits had practically no impact on the group without a disability, but impacted those with a disability, supposing a disadvantage of their entry and stability in the labor market. The same applies to the age variable: no effect was detected among graduates with or without disabilities, although the age group over 34 had a lower chance of success in the employment world. Knowing several languages had a moderate influence. Finally, regarding gender, among the group with disabilities, women were more likely to succeed in entering the labor market, whereas among the group of people without disabilities it was the other way around: men were favored. Therefore, having a disability has a negative impact on all the characteristics with which we have defined success in entering the labor market. Thus, according to the analysis conducted, graduates with some type of disability have half the possibility of working with respect to graduates who do not have a disability, their employment stability and salary are reduced by about 25%, and they have a 40% more chance of working part time.

The analysis shows the factors that influence the labor market insertion of graduates with disabilities, and this relationship with the group of people without disabilities. Future research could consider different types of disabilities to obtain a better characterization of the employment situation. In any case, the results obtained can be used for future action by public authorities, since policies could be implemented to encourage and improve the employability of people with disabilities, considering the variables that influence their labor market insertion.

## Figures and Tables

**Table 1:** Success of labor market insertion of graduates in Spain, 2014–2019.

		2014		2019	
		Not Disab	Disab	Not Disab	Disab
EMP_SIT	Working	80.8%	75.7%	86.2%	75.4%
	Not working	19.2%	24.3%	13.8%	24.6%
EMP_STA	Permanent	46.3%	46.4%	58.2%	53.2%
	Temporary	53.7%	53.6%	41.8%	46.8%
INC_Q	High inc.	42.3%	41.9%	30.3%	26.8%
	Low inc.	57.7%	58.1%	69.7%	73.2%
EMP_ADE	Higher edu. required	74.8%	68.2%	68.3%	63.3%
	Not required	25.2%	31.8%	31.7%	36.7%
WOR_HOU	Full time	76.5%	78.7%	74.3%	64.5%
	Part time	23.5%	21.3%	25.7%	35.5%

Source: Own elaboration based on data from the Spanish National Institute of Statistics.

**Table 2:** Effect of disability on the success of employment in Spain.

	Success in Labor Market Insertion									
	EMP-SIT		EMP-STA		INC_Q		EMP_ADE		WOR_HOU	
Factors	Sig.	Exp(B)	Sig.	Exp(B)	Sig.	Exp(B)	Sig.	Exp(B)	Sig.	Exp(B)
Work	0	2.006	0	1.697	0	1.424	0	1.353	0	1.538
Scholarship	0.708	1.007	0	0.946	0.08	1.026	0.07	1.027	0	1.123
Mobility	0	1.142	0	1.325	0	1.096	0	1.263	0	1.172
Gender	0	0.847	0	0.698	0	0.746	0	0.915	0	0.66
Age	0	0.921	0	1.221	0	1.163	0	0.886	0	1.045
ICT	0	1.374	0	1.414	0	1.152	0	1.332	0	1.541
Languages	0.001	0.975	0	0.968	0	0.938	0	1.072	0	0.977
DISAB	**0**	**0.504**	**0**	**0.736**	**0**	**0.762**	**0.004**	**0.882**	**0**	**0.607**
Constant	0	3.209	0	0.483	0	0.269	0	1.17	0	1.19

Source: Own elaboration based on data from the Spanish National Institute of Statistics.

**Table 3:** Effect of external factors on the success of labor market insertion of graduates in Spain.

		Success in Labor Market Insertion									
		EMP-SIT		EMP-STA		INC_Q		EMP_ADE		WOR_HOU	
Factors		Sig.	Exp(B)	Sig.	Exp(B)	Sig.	Exp(B)	Sig.	Exp(B)	Sig.	Exp(B)
Work	Disab	0	6.074	0	3.914	0	2.166	0	2.686	0	3.834
	Not Disab	0	1.974	0	1.683	0	1.417	0	1.342	0	1.521
Scholarship	Disab	0	0.303	0.003	0.548	0.013	1.73	0.001	0.506	0.432	0.85
	Not Disab	0.461	1.014	0	0.948	0.107	1.024	0.04	1.03	0	1.127
Mobility	Disab	0	9.033	0	4.339	0.002	0.443	0	3.535	0.711	1.084
	Not Disab	0	1.131	0	1.318	0	1.1	0	1.257	0	1.173
Gender	Disab	0	2.193	0.588	1.052	0	1.632	0	1.421	0	1.449
	Not Disab	0	0.834	0	0.694	0	0.74	0	0.91	0	0.652
Age	Disab	0	0.326	0.008	0.847	0.001	1.264	0	0.525	0	0.499
	Not Disab	0	0.935	0	1.226	0	1.162	0	0.891	0	1.055
ICT	Disab	0	2.435	0	1.809	0	1.72	0	1.724	0	1.986
	Not Disab	0	1.362	0	1.41	0	1.147	0	1.328	0	1.534
Languages	Disab	0.002	0.812	0	0.731	0.002	0.816	0.001	0.819	0.315	0.941
	Not Disab	0.003	0.978	0	0.971	0	0.939	0	1.075	0	0.979
Constant	Disab	0	2.876	0	0.355	0	0.045	0.051	1.637	0.783	1.074
	Not Disab	0	3.221	0	0.484	0	0.272	0	1.68	0	1.194

Source: Own elaboration based on data from the Spanish National Institute of Statistics.

**Table 4:** Influence of educational factors on the success of labor market insertion of graduates with disabilities in Spain.

	EMP_SIT	EMP_STA	INC_Q	EMP_ADE	WOR_HOU
Work	**0.653**	**0.797**	**0.836**	**0.561**	**0.781**
ICT	**0.575**	**0.393**	**0.419**	**0.581**	**0.676**
Mobility	**0.283**	0.163	−0.411	**0.370**	−0.132
Scholarship	0.054	0.021	−0.118	0.092	−0.040
Languages	−0.071	−0.234	−0.367	−0.123	−0.019
Correct classified	75.9%	66.5%	73.0%	65.5%	67.3%

Source: Own elaboration based on data from the Spanish National Institute of Statistics.

## Data Availability

Publicly available datasets were analyzed in this study. This data can be found here: https://ine.es/dyngs/INEbase/en/operacion.htm?c=Estadistica_C&cid=1254736176991&menu=resultados&idp=1254735573113#!tabs-1254736195339 (accessed on 15 December 2021).
